# HCV Induces the Expression of Rubicon and UVRAG to Temporally Regulate the Maturation of Autophagosomes and Viral Replication

**DOI:** 10.1371/journal.ppat.1004764

**Published:** 2015-03-25

**Authors:** Linya Wang, Yongjun Tian, Jing-hsiung James Ou

**Affiliations:** Department of Molecular Microbiology and Immunology, University of Southern California, Keck School of Medicine, Los Angeles, California, United States of America; University of Alabama at Birmingham School of Medicine, UNITED STATES

## Abstract

Hepatitis C virus (HCV) induces autophagy to enhance its replication. However, how HCV regulates the autophagic pathway remains largely unclear. In this report, we demonstrated that HCV infection could induce the expression of Rubicon and UVRAG, which inhibited and stimulated the maturation of autophagosomes, respectively. The induction of Rubicon by HCV was prompt whereas the induction of UVRAG was delayed, resulting in the accumulation of autophagosomes in the early time points of viral infection. The role of Rubicon in inhibiting the maturation of autophagosomes in HCV-infected cells was confirmed by siRNA knockdown and the over-expression of Rubicon, which enhanced and suppressed the maturation of autophagosomes, respectively. Rubicon played a positive role in HCV replication, as the suppression of its expression reduced HCV replication and its over-expression enhanced HCV replication. In contrast, the over-expression of UVRAG facilitated the maturation of autophagosomes and suppressed HCV replication. The HCV subgenomic RNA replicon, which expressed only the nonstructural proteins, could also induce the expression of Rubicon and the accumulation of autophagosomes. Further analysis indicated that the HCV NS4B protein was sufficient to induce Rubicon and autophagosomes. Our results thus indicated that HCV, by differentially inducing the expression of Rubicon and UVRAG, temporally regulated the autophagic flux to enhance its replication.

## Author Summary

HCV induces autophagy to benefit its replication. In this report, we demonstrated that HCV infection could induce the expression of both Rubicon and UVRAG, which inhibited and stimulated the maturation of autophagosomes, respectively. The induction of Rubicon by HCV was prompt whereas the induction of UVRAG was delayed, resulting in the accumulation of autophagosomes in the early time points of viral infection. Rubicon positively regulated HCV replication, apparently by increasing the pool of autophagosomes, which could serve as the sites for HCV RNA replication. On the contrary, UVRAG, which reduced the pool of autophagosomes, inhibited HCV replication if it was over-expressed prior to HCV infection. The analysis of HCV genes indicated that the HCV NS4B protein was sufficient to induce the expression of Rubicon and the accumulation of autophagosomes. Our results thus revealed a novel mechanism used by a virus to temporally regulate the autophagic flux for its replication.

## Introduction

Hepatitis C virus (HCV) is an important human pathogen that can cause severe liver diseases including cirrhosis and hepatocellular carcinoma. It belongs to the flavivirus family and has a 9.6 Kb positive-stranded RNA genome. This genome encodes a polyprotein with a length of slightly more than 3,000 amino acids. The translation of the HCV polyprotein is mediated by an internal ribosomal entry site (IRES) that comprises most of the 5’-untranslated region and the first few codons of the polyprotein coding sequence. After its synthesis, the HCV polyprotein is processed into structural and nonstructural proteins by cellular and viral proteases [1]. The nonstructural proteins NS3, NS4A, NS4B, NS5A and NS5B are required and sufficient for viral RNA replication [2].

HCV is a hepatotropic virus. It can induce autophagy in its host cells to enhance its replication [3–12]. Autophagy is a catabolic process by which cells remove protein aggregates and damaged organelles for recycling. This process begins by the formation of membrane crescents termed phagophores in the cytosol. The edges of these phagophores will subsequently extend to sequester part of the cytoplasm, leading to the formation of enclosed double-membrane vesicles, known as autophagosomes. Autophagosomes mature by fusing with lysosomes to form autolysosomes, in which the cargos of autophagosomes are digested by lysosomal enzymes for recycling [13]. Autophagy occurs at a basal level in cells during normal conditions and is important for maintaining cellular homeostasis. It can also be induced by stress, such as nutrient starvation.

Many protein factors that are important for autophagy have been identified. Class III phosphatidylinositol-3-kinase (PI3KC3) is one of these factors. It catalyzes the formation of phosphatidylinositol-3-phosphate (PI3P) and is important for the initiation of autophagy [14]. PI3KC3 consists of three core components, hVps34, p150 and Beclin-1 [14,15]. This core complex is associated with two mutually exclusive factors Atg14L and UVRAG [16–18]. Atg14L is important for autophagosomal membrane nucleation and expansion, whereas UVRAG plays a more important role in the maturation of autophagosomes [19]. UVRAG can bind to the class C Vps/HOPS complex, which is a guanine nucleotide exchange factor for the small GTPase Rab7, to activate Rab7 and stimulate the fusion between autophagosomes and lysosomes [20]. The UVRAG activity is negatively regulated by Rubicon (RUN domain and cysteine-rich domain containing, Beclin 1-interacting protein), which binds to the UVRAG-PI3KC3 complex to inhibit the maturation of autophagosomes [21]. Rubicon can also sequester UVRAG from C-Vps/HOPS and inhibit the activation of Rab7 [22]. Another factor that is important for autophagy is the microtubule-associated protein light-chain 3 (LC3). LC3 is a cytosolic protein. During autophagy, it is covalently linked by Atg3, Atg4 and Atg7 to phosphatidylethanolamine (PE), a phospholipid. This lipidation allows LC3 to localize to autophagosomal membranes. LC3 is de-lipidated by Atg4 after the maturation of autophagosomes and released back into the cytosol. It can also be degraded by lysosomal enzymes, if it is localized to the inner membrane of autophagosomes [13]. The lipidation of LC3 and its localization to autophagosomes are often used as markers for autophagy.

HCV can induce the accumulation of autophagosomes and use autophagosomal membranes as the site for its RNA replication [5,12]. However, there are controversies regarding whether HCV can efficiently induce the fusion between autophagosomes and lysosomes [5,7,9,11,23–25]. In this report, we investigated the mechanism of HCV-induced autophagy. Our results indicated that HCV could induce the expression of both Rubicon and UVRAG. The induction of Rubicon was prompt whereas the induction of UVRAG was delayed, leading to the delayed maturation and the accumulation of autophagosomes in the early time points of HCV infection. Our results thus indicated that HCV, by differentially regulating the expression of Rubicon and UVRAG, temporally regulated the autophagic flux to enhance its replication.

## Results

### Delayed maturation of autophagosomes in HCV-infected cells

**Fig 1:**
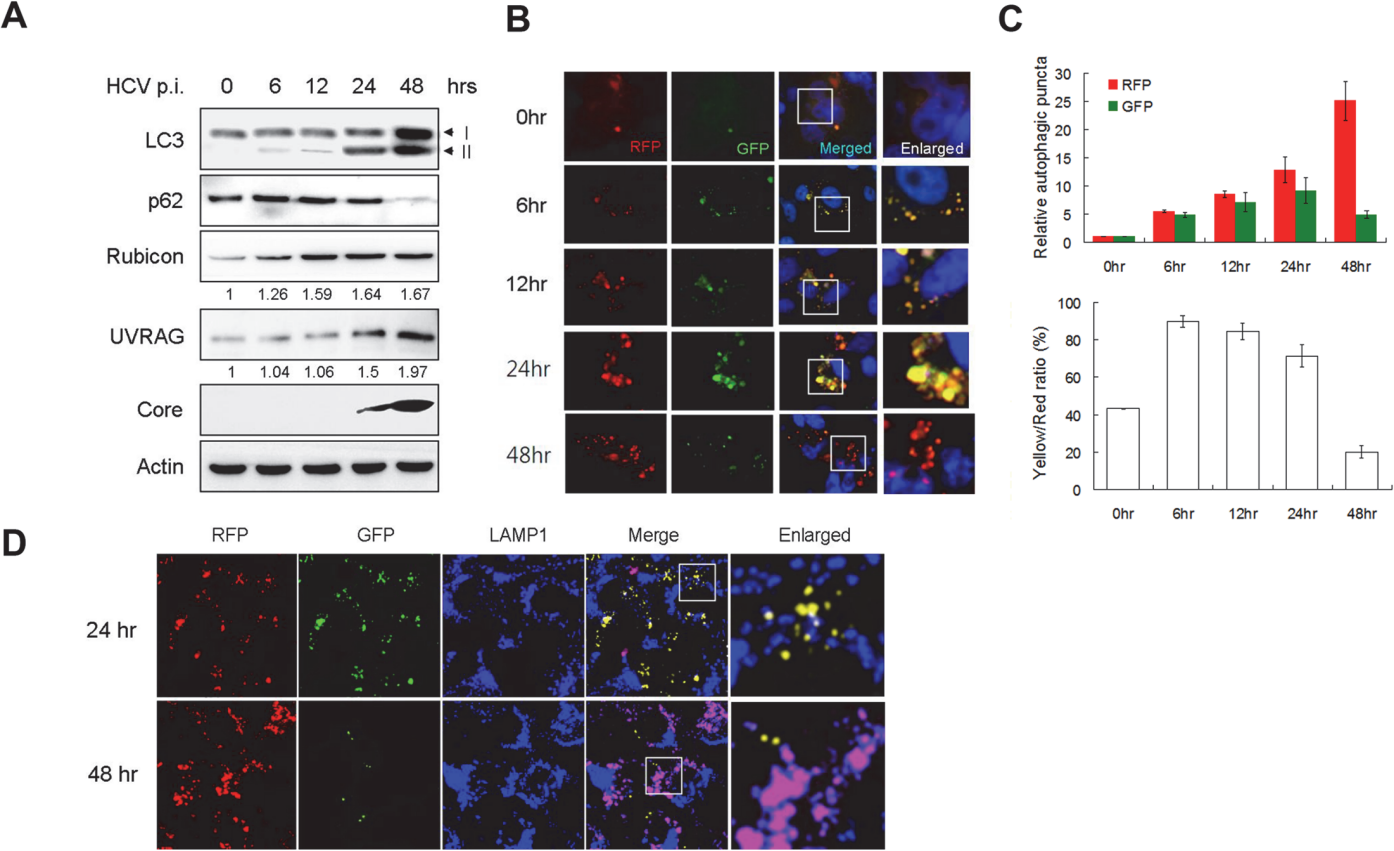
Analysis of autophagosomal maturation in HCV-infected cells. (A) Western-blot analysis of HCV-infected cells at various time-points post-infection (p.i.) (m.o.i. = 1). Numbers under Rubicon and UVRAG panels indicate their protein levels at different time points relative to their levels in mock-infected cells (i.e., 0 hour p.i.). (B) Fluorescence imaging of HCV infected cells. Stable Huh7.5 cells that expressed mRFP-GFP-LC3 were infected with HCV and fixed at the time points indicated for the analysis of RFP and GFP puncta. Nuclei were stained with DAPI. The boxed areas were enlarged and shown to the right. (C) Upper panel, levels of RFP and GFP puncta after HCV infection relative to those in mock-infected cells, which were arbitrarily defined as 1. Lower panel, percentages of RFP puncta that were positive for GFP (i.e., Yellow/Red ratio). The results represent the average of >50 cells that were analyzed. (D) Fluorescence imaging of RFP, GFP and LAMP1 in HCV-infected cells expressing mRFP-GFP-LC3. The lysosomal marker LAMP1 was stained in blue color. DAPI was not used to stain the nuclei. The colocalization of RFP and GFP will generate the yellow color, and the colocalization of RFP with LAMP without GFP will generate the purple color. The boxed areas were enlarged and shown to the right.

To understand how HCV induces autophagy, we infected Huh7.5 cells with a variant of the HCV JFH1 isolate. This variant replicated more efficiently than its parental virus [26,27], and with an m.o.i. of 1, the HCV core protein could be detected in >90% of the cells by day 2 post-infection (S1A Fig.), indicating an efficient viral replication and propagation. Cells were lysed at different time points after infection for Western-blot analysis. As shown in Fig. 1A, the level of lipidated LC3 (i.e., LC3-II) was slightly increased at 6 hours and 12 hours post-infection and significantly increased at 24 hours and 48 hours post-infection when the intracellular HCV core protein also became apparent. We also analyzed p62, which is a protein removed by autophagy and often used as a marker for measuring the autophagic protein degradation rate [28]. Interestingly, in spite of the induction of LC3 lipidation during the first 24 hours after infection, there was no reduction of the p62 level during this time period. Rather, a light increase of p62 was observed at 6 and 12 hours post-infection (Fig. 1A). The level of p62 became almost undetectable at 48 hours post-infection. These results indicated that, although HCV was able to induce an autophagic response as early as 6 hours post-infection, it apparently was not able to induce efficient autophagic protein degradation and complete the autophagic process until 48 hours post-infection. Similar results were obtained when Huh7 cells, the parental cell line of Huh7.5, were used for the infection studies (S2 Fig.). HCV was not able to induce the degradation of p62 in Huh7 cells until 48 hours post-infection.

To investigate why the autophagic protein degradation appeared to be inefficient in the first 24 hours of HCV infection, we infected stable Huh7.5 cells that expressed the mRFP-GFP-LC3 tripartite fusion protein with HCV. This fusion protein is localized diffusely in the cytosol, but upon the induction of autophagy, it is localized to autophagic vacuoles due to the lipidation of LC3. As the red fluorescence signal produced by mRFP of this fusion protein is not sensitive to acid whereas the green fluorescence signal produced by GFP is [28], this fusion protein served as a convenient tool for us to distinguish between autophagosomes, which is not acidic, and autolysosomes, which is acidic. Cells were fixed at different time points after infection for the analysis of RFP and GFP signals. As shown in Fig. 1B and the upper panel of Fig. 1C, the number of RFP puncta was low in mock-infected cells and increased over time after HCV infection. When the GFP puncta was analyzed, its level was also increased by HCV progressively during the first 24 hours of infection. However, its level was significantly reduced at 48 hours post-infection. When both RFP and GFP signals were merged, approximately 40% of RFP puncta in mock-infected cells were positive for GFP (Fig. 1C, lower panel), indicating that they were autophagosomes, with the remaining RFP puncta being autolysosomes [28]. However, at 6, 12 and 24 hours post-HCV infection, between 70–90% of RFP puncta were positive for GFP, indicating a disproportionally higher level of autophagosomes than autolysosomes during this time period of HCV infection. The percentage of RFP puncta that was also positive for GFP dropped significantly to about 20% at 48 hours post-infection, indicating an efficient maturation of autophagosomes and also providing an explanation to the loss of p62 at this time point (Fig. 1C). To ensure that these cells were indeed infected by HCV, we also stained the cells with the anti-HCV core antibody. In agreement with the results shown in S1A Fig., essentially all of the cells were positive for the HCV core protein (S1B Fig.), and, the same as the results shown in Fig. 1B, most of the RFP puncta were positive for GFP at 24 hours post-infection, but only a few of the RFP puncta were positive for GFP at 48 hours. To confirm the results shown in Fig. 1B and S1B Fig., we also stained the cells with the antibody directed against LAMP1, a lysosomal marker. As shown in Fig. 1D, most RFP puncta were positive for GFP (i.e., yellow puncta after merging of the images) and only a few of them colocalized with LAMP1 (i.e., purple puncta after merging of the images) at 24 hours post-infection. In contrast, most RFP puncta colocalized with LAMP1 and were negative for GFP at 48 hours post-infection. These results were in agreement with the results shown in Fig. 1B and demonstrated that the fusion between autophagosomes and lysosomes were inefficient at 24 hours post-infection but was highly efficient at 48 hours.

### Differential induction of Rubicon and UVRAG by HCV

Rubicon and UVRAG have antagonistic activities in the regulation of maturation of autophagosomes [21]. To understand the molecular mechanism that regulates the maturation of autophagosomes in HCV infected cells, we analyzed the expression levels of Rubicon and UVRAG in Huh7.5 cells at various time points after HCV infection. As shown in Fig. 1A, the level of Rubicon increased at 6 hours post-infection, coincided with the first observed increase of lipidated LC3. The level of Rubicon further increased at 12 hours post-infection and remained roughly at that level up to 48 hours post-infection. The level of UVRAG was also increased by HCV, although this increase was not prominent until 48 hours post-infection (Fig. 1A). When Huh7 cells were used for the infection studies, a similar result was obtained. HCV significantly increased the level of Rubicon at 24 and 48 hours post-infection. However, it did not significantly increase the level of UVRAG until 48 hours post-infection (S2 Fig.). These results raised the possibility that the inefficient maturation of autophagosomes in the first 24 hours of HCV infection might be due the induction of Rubicon, which inhibited the maturation of autophagosomes. This inhibitory effect of Rubicon was then overcome by the significant increase of the UVRAG level at 48 hours post-infection. To understand how HCV induced the expression of Rubicon and UVRAG, we analyzed the RNA levels of Rubicon and UVRAG in HCV-infected Huh7.5 cells by semi-quantitative RT-PCR. As shown in S3 Fig., HCV increased both Rubicon and UVRAG RNA levels at both 24 and 48 hours post-infection, and those increases were more or less in good correlation with the increases of their protein levels at these two time points, indicating that HCV most likely induced the expression of these two proteins at the transcriptional level.

### Suppression of autophagosomal maturation and enhancement of viral replication by Rubicon in HCV-infected cells

**Fig 2:**
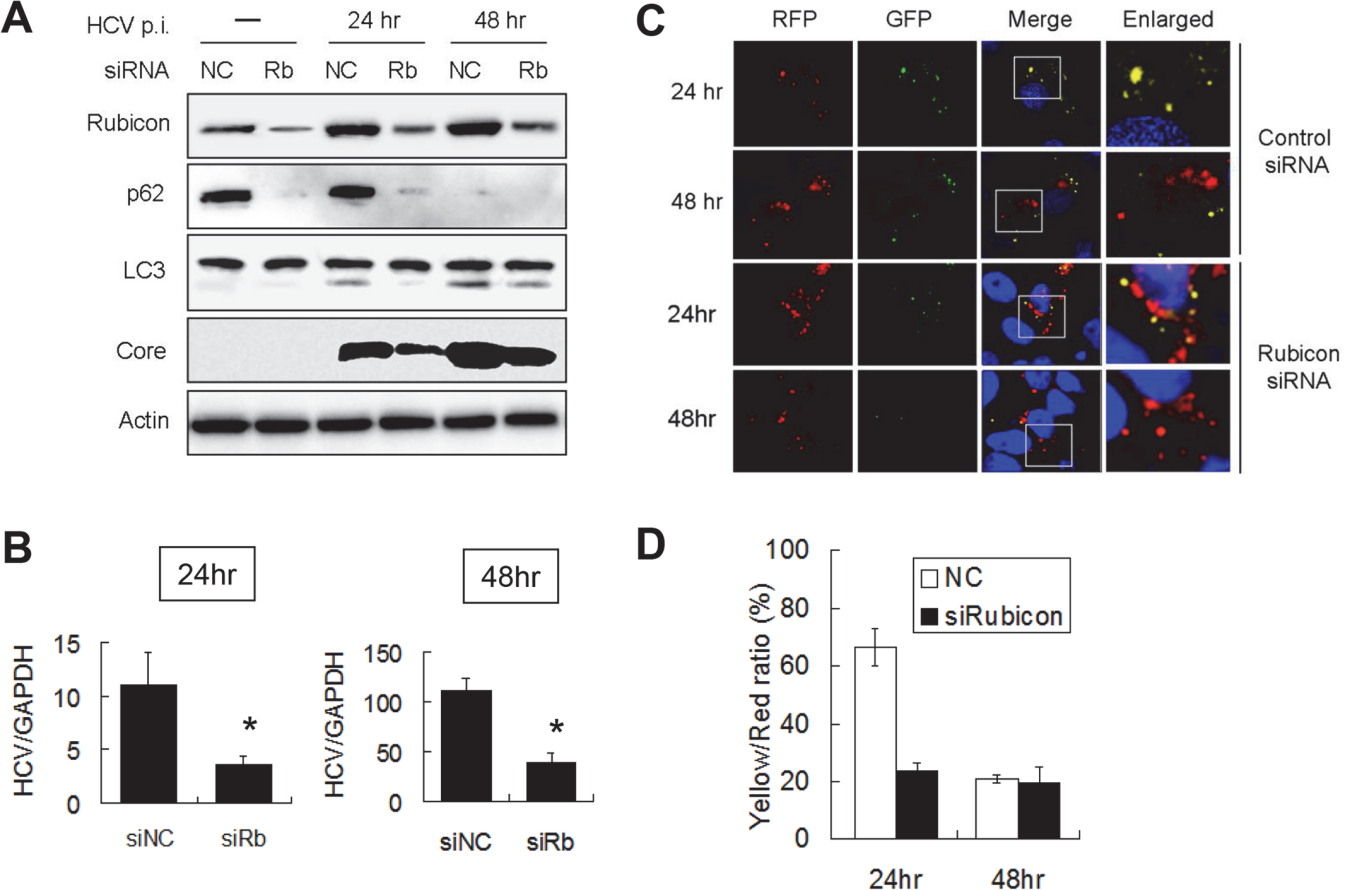
Suppression of Rubicon expression enhanced the maturation of autophagosomes and inhibited HCV RNA replication. Huh7.5 cells were transfected with the negative control siRNA (NC) or the Rubicon (Rb) siRNA for 48 hours and then infected with 1 m.o.i. of HCV. (A) Western-blot analysis of cell lysates at different time points after HCV infection. Actin served as the loading control. (B) Real-time RT-PCR analysis of HCV RNA at 24 and 48 hours post-infection. *, p < 0.05. siNC, negative control siRNA; siRb, Rubicon siRNA. (C) Fluorescence imaging of RFP and GFP puncta in cells transfected with the control siRNA (top two panels) or the Rubicon siRNA (bottom two panels). Cells were fixed at 24 and 48 hours after HCV infection for the analysis. Boxed areas in merged images are enlarged and shown to the right. (D) Percentages of RFP puncta that were also positive for GFP in Huh7.5 cells treated with either the control siRNA or the Rubicon siRNA. The results represent the average of >50 cells.

To test whether Rubicon indeed negatively regulated the maturation of autophagosomes in the first 24 hours after HCV infection, we performed the siRNA knockdown experiment to suppress the expression of Rubicon in Huh7.5 cells, which were then infected with HCV for either 24 hours or 48 hours. As shown in Fig. 2A, the suppression of Rubicon expression reduced the p62 level in mock-infected and HCV-infected cells, indicating a role of Rubicon in the inhibition of autophagic protein degradation. When LC3 was analyzed, the effect of Rubicon siRNA on LC3-II was inapparent in mock-infected cells due to the low LC3-II level. However, the Rubicon siRNA reduced the LC3-II level in HCV-infected cells at both 24 and 48 hours post-infection. As LC3-II is either delipidated or degraded by lysosomal enzymes after the maturation of autophagosomes, the reduction of its level as well as the reduction of p62 by the Rubicon siRNA is in support of a role of Rubicon in inhibiting the maturation of autophagosomes. The inhibition of Rubicon expression also reduced the HCV core protein level at both 24 and 48 hours post-infection, suggesting a positive role of Rubicon in HCV replication (Fig. 2A). To determine whether Rubicon indeed played a positive role in HCV replication, we also analyzed the effect of Rubicon knockdown on HCV RNA replication by real-time RT-PCR. As shown in Fig. 2B, Rubicon siRNA significantly reduced the HCV RNA level at both 24 and 48 hours post-infection.

To further confirm the role of Rubicon in the maturation of autophagosomes, we transfected stable Huh7.5 cells that expressed mRFP-GFP-LC3 with the control or the Rubicon siRNA followed by HCV infection. As shown in Fig. 2C, the knockdown of Rubicon significantly diminished the signals of GFP puncta at 24 hours post-infection. The quantitative analysis of RFP and GFP puncta revealed that approximately 70% of RFP puncta were positive for GFP at this time point in the control siRNA transfected cells, but this percentage was reduced to about 25% by Rubicon siRNA (Fig. 2D). These results indicated that the depletion of Rubicon could facilitate the maturation of autophagosomes. The depletion had little effect on autophagosomes at 48 hours post-infection (Fig. 2D), presumably because the maturation of autophagosomes at this time point was already highly efficient.

**Fig 3:**
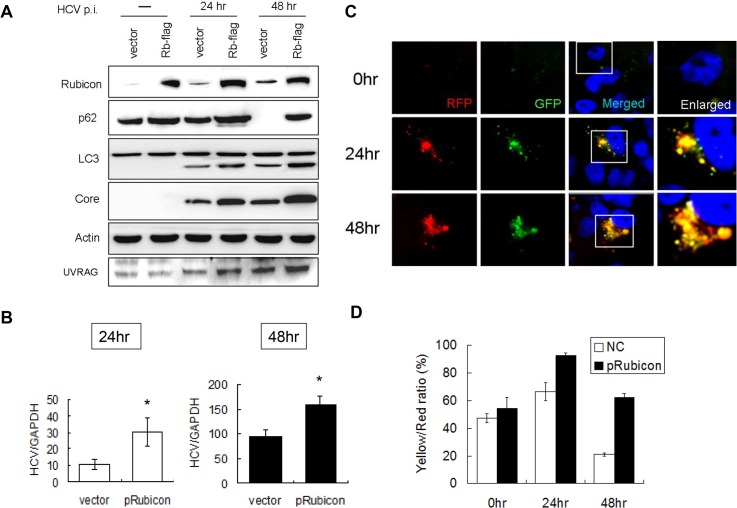
Over-expression of Rubicon inhibited the maturation of autophagosomes and enhanced HCV RNA replication. Huh7.5 cells were transfected with the control vector or the Flag-tagged Rubicon expression plasmid for 24 hours followed by infection with HCV. (A) Western-blot analysis of cell lysates at different time points after infection. Mock-infected cells were lysed at 48 hours post-transfection. (B) Real-time RT-PCR analysis of HCV RNA at 24 and 48 hours post-infection. *, p < 0.05. (C) RFP and GFP puncta in cells with the over-expression of Rubicon at different time points after HCV infection. Merged images are shown to the right. (D) Percentages of RFP puncta that were also positive for GFP in Huh7.5 cells transfected with either the control vector or the Rubicon expression plasmid. The results represent the average of >50 cells.

If Rubicon indeed negatively regulated the maturation of autophagosomes in HCV-infected cells, then its over-expression using an expression vector should further inhibit the maturation of autophagosomes, even at 48 hours post-infection. To test this possibility, we transfected Huh7.5 cells with an expression plasmid of Flag-tagged Rubicon. The transfection efficiency was determined by immunostaining, which revealed that most cells were positive for the Flag-tagged Rubicon (S4 Fig.). The transfected cells were then infected with HCV. As shown in Fig. 3A, the over-expression of Rubicon significantly increased the Rubicon level and marginally increased the level of UVRAG. This over-expression of Rubicon increased the p62 level in HCV-infected cells at both 24 and 48 hours post-infection. It also slightly increased the LC3-II protein level. These increases of p62 and LC3-II protein levels were consistent with the reduction of the maturation efficiency of autophagosomes. In contrast to Rubicon knockdown, which reduced HCV core protein and RNA levels (Fig. 2A and 2B), the over-expression of Rubicon increased the HCV core protein level (Fig. 3A) as well as the HCV RNA level (Fig. 3B). These results again supported a positive role of Rubicon in HCV replication.

To confirm the role of Rubicon in autophagosomal maturation, we also expressed Flag-tagged Rubicon in stable mRFP-GFP-LC3 cells followed by infection with HCV. As shown in Fig. 3C, the over-expression of Rubicon increased the level of GFP puncta at 48 hours post-infection. The quantitative analysis indicated that the ratio of RFP puncta that were also positive for GFP was increased from 20% to 60% at this time point (Fig. 3D). The results shown in Fig. 2 and 3 thus confirmed that Rubicon induced by HCV played an important role in inhibiting the maturation of autophagosomes in HCV-infected cells and that Rubicon played a positive role in HCV replication.

### Enhancement of autophagosomal maturation and suppression of viral replication by UVRAG in HCV-infected cells

**Fig 4:**
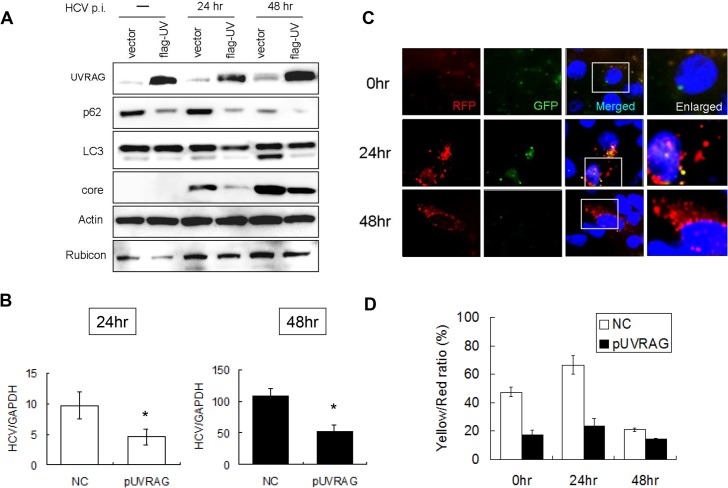
Over-expression of UVRAG enhanced the maturation of autophagosomes and reduced HCV RNA replication. Huh7.5 cells were transfected with the control vector or the Flag-tagged UVRAG expression plasmid for 24 hours followed by infection with HCV. (A) Western-blot analysis of cell lysates at different time points after infection. Mock-infected cells were lysed at 48 hours post-transfection. (B) Real-time RT-PCR analysis of HCV RNA at 24 and 48 hours post-infection. *, *p* < 0.05. (C) RFP and GFP puncta in cells with the over-expression of UVRAG at different time points after HCV infection. Merged images are shown to the right. (D) Percentages of RFP puncta that were also positive for GFP in Huh7.5 cells transfected with either the control vector or the UVRAG expression plasmid. The results represent the average of >50 cells.

In contrast to the first 24 hours of infection, the maturation of autophagosomes and the autophagic protein degradation were efficient at 48 hours post-infection. As there was a significant increase of the UVRAG level at 48 hours (Fig. 1A), it is conceivable that the efficient maturation of autophagosomes at this time point was due to the significant rise of the UVRAG level, which overcame the inhibitory effect of Rubicon. To test this possibility, we first conducted the siRNA knockdown experiment to determine whether the inhibition of UVRAG expression would prevent the maturation of autophagosomes at 48 hours post-infection. Unfortunately, this study generated inconclusive results, as the inhibition of UVRAG expression significantly reduced the HCV infectivity (S5A Fig.), likely due to its essential role in endocytic membrane trafficking and hence HCV entry [22]. We therefore chose to test whether the over-expression of UVRAG could facilitate the maturation of autophagosomes in HCV-infected cells by expressing Flag-tagged UVRAG in Huh7.5 cells followed by HCV infection. The transfection efficiency of Flag-tagged UVRAG plasmid was similarly monitored by immunofluorescence staining, which revealed that most cells were positive for Flag-tagged UVRAG (S4 Fig.). Huh7.5 cells transfected with either the control vector or the Flag-tagged UVRAG expression plasmid were then infected with HCV. As shown in Fig. 4A, the over-expression of UVRAG reduced the levels of p62, LC3-II and Rubicon in mock-infected and HCV-infected cells. It also reduced the fraction of RFP puncta that were positive for GFP (Fig. 4C and 4D). These results supported a role of UVRAG in facilitating the maturation of autophagosomes in HCV-infected cells. The over-expression of UVRAG reduced the HCV core protein and RNA levels (Fig. 4A and 4B), indicating a negative role of UVRAG in HCV replication.

### Opposite effects of Rubicon and UVRAG on HCV replication

**Fig 5:**
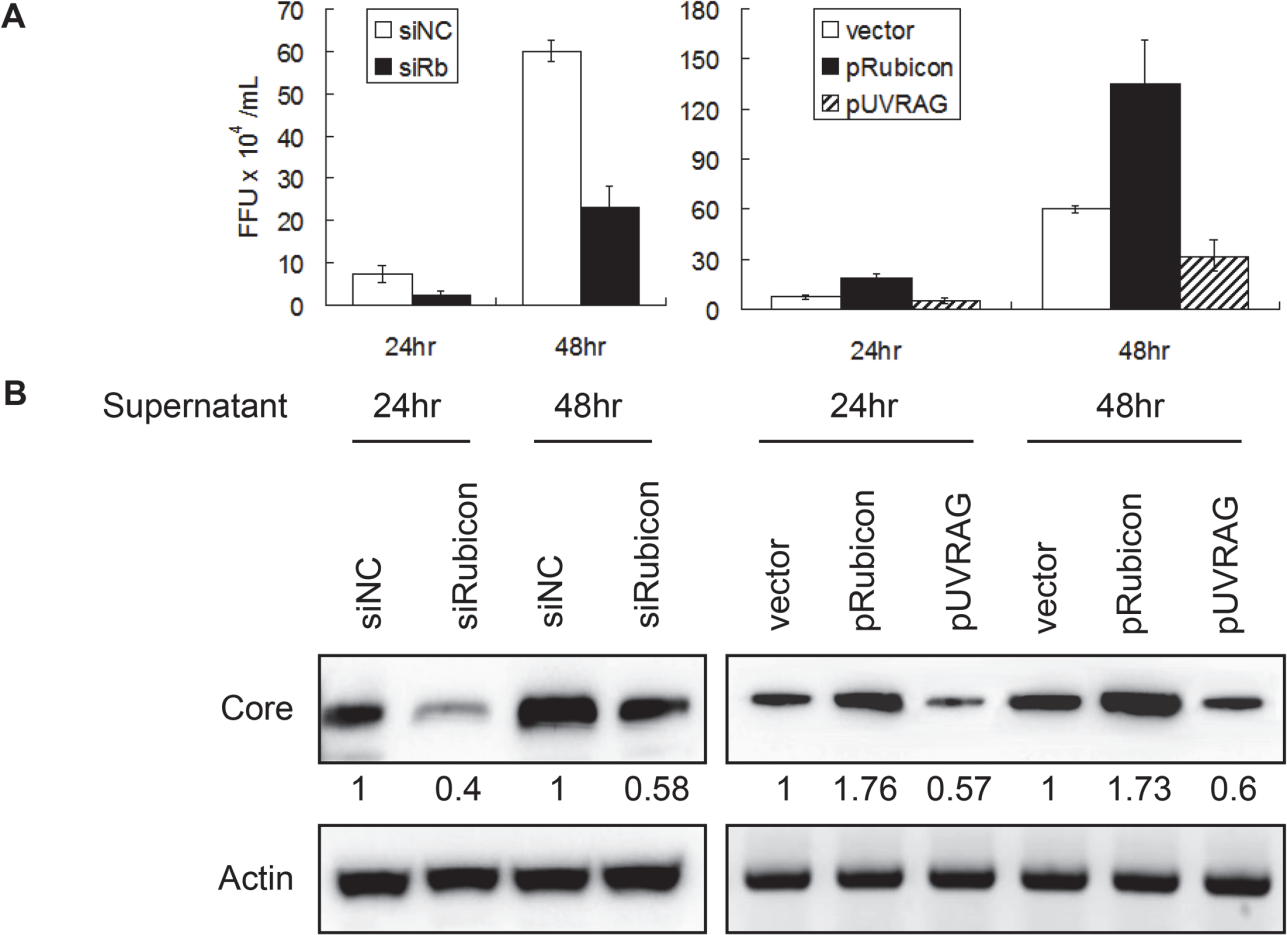
Analysis of the effects of Rubicon and UVRAG on HCV yield. Cells transfected with the control siRNA (siNC), the Rubicon siRNA (siRb), the Rubicon expression plasmid (pRubicon) or the UVRAG expression plasmid (pUVRAG) were infected with HCV (m.o.i. = 1). The incubation media were harvested at 24 and 48 hours post-infection and used to infect naïve Huh7.5 cells. Cells were either fixed and stained for the HCV core protein for the determination of viral titers (A) or lysed for Western-blot analysis of the HCV core protein (B) two days after infection. The results shown in (A) represent the average of three independent experiments, and the numbers under the core protein panels in (B) indicate the relative core protein levels, with the core protein level of control siRNA transfected cells arbitrarily defined as 1. Actin served as the loading control in (B).

The results described above indicated that Rubicon and UVRAG had opposite effects on HCV core protein and RNA levels in cells. To determine whether Rubicon and UVRAG also affected the yield of progeny virus, we harvested the incubation media of HCV-infected cells at 24 and 48 hours post-infection and used them to infect naive cells for the determination of viral titers and for Western-blot analysis of the HCV core protein. As shown in Fig. 5, cells treated with the Rubicon siRNA produced a lower viral titer (Fig. 5A) and a lower level of the core protein (Fig. 5B) than the cells treated with the control siRNA, regardless of whether it was at 24 or 48 hours post-infection. On the contrary, cells with the over-expression of Rubicon produced a higher viral titer (Fig. 5A) and a higher level of the core protein (Fig. 5B) than the cells transfected with the control vector. These results, together with the observations that Rubicon increased the intracellular HCV RNA level (Figs. 2B and 3B), indicated that Rubicon increased the overall HCV replication efficiency and virus yield. In contrast to Rubicon, the over-expression of UVRAG produced a lower HCV titer (Fig. 5A) and a lower level of the core protein (Fig. 5B), confirming a negative role of UVRAG in HCV replication.

### Regulation of autophagosomal maturation and HCV RNA replication by Rubicon and UVRAG in HCV subgenomic RNA replicon cells

**Fig 6:**
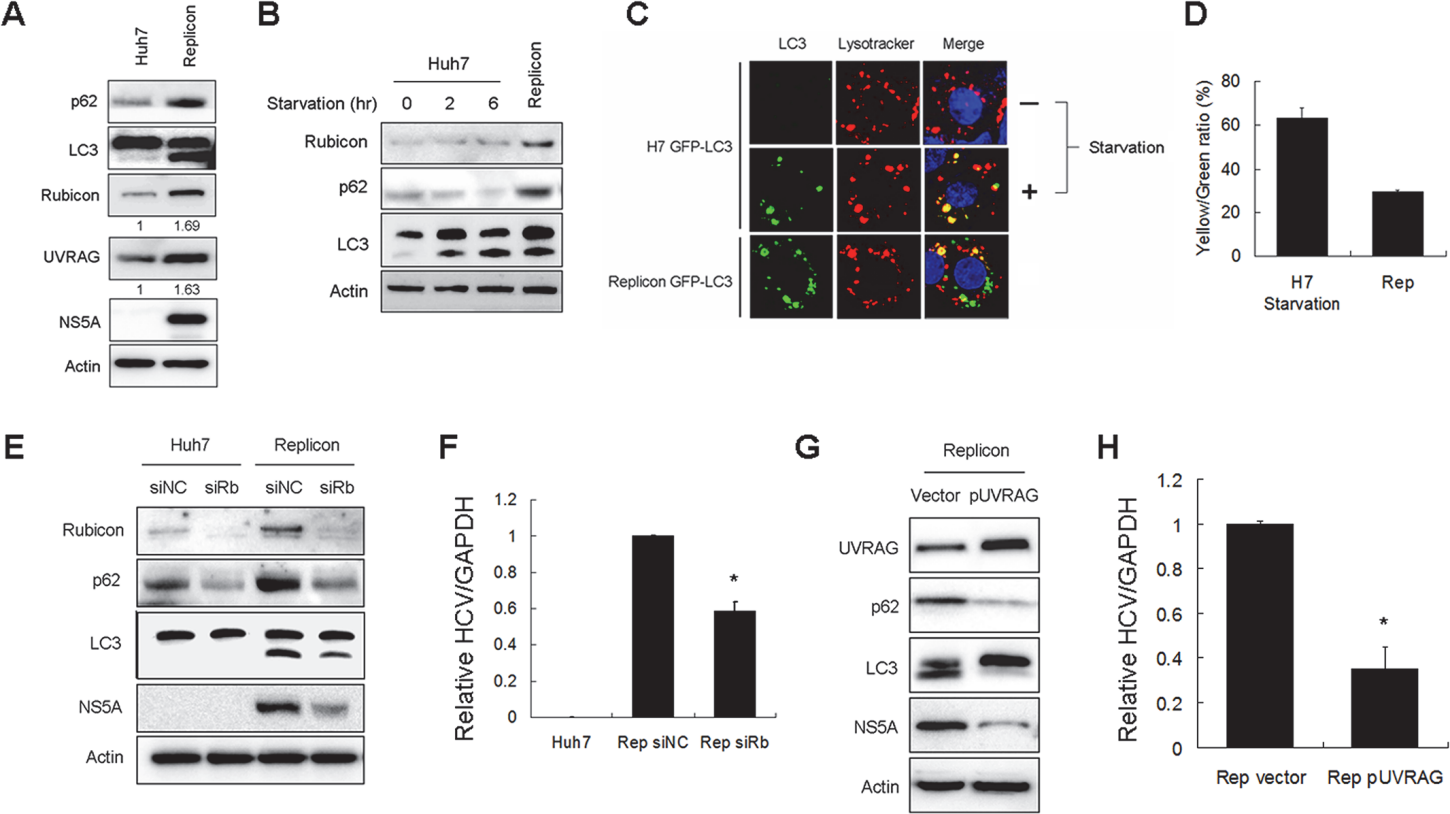
Effects of Rubicon on autophagosomal maturation and HCV RNA replication in HCV replicon cells. (A) Increase of Rubicon, UVRAG, p62 and LC3-II in HCV subgenomic RNA replicon cells. Actin served as the loading control. Numbers under Rubicon and UVRAG indicate the protein levels of Rubicon and UVRAG in replicon cells relative to their levels in control Huh7 cells. (B) Effects of nutrient starvation on Rubicon, p62 and LC3 in Huh7 cells. Huh7 cells were nutrient-starved for 2 or 6 hours as indicated and lysed for Western-blot analysis. The replicon cells were used as the control for comparison. (C) Colocalization analysis of GFP-LC3 puncta and lysosomes. Stable Huh7 cells that expressed GFP-LC3 were nutrient-starved for 2 hours and stained with Lysotracker-red for lysosomes. The HCV replicon cells were also stained with Lysotracker-red for comparison. (D) Colocalization efficiency of GFP puncta with Lysotracker-red shown in (C). The results represent the average of >30 cells. (E) Effect of Rubicon knockdown on parental Huh7 cells and HCV replicon cells. Huh7 cells and HCV replicon cells were treated with the control siRNA or the Rubicon siRNA for two days. Cells were then lysed for Western-blot analysis. (F) Relative HCV RNA levels as measured by real-time RT-PCR. HCV replicon cells treated with either the control siRNA or the Rubicon siRNA for two days were lysed for quantification of HCV RNA by real-time RT-PCR. (G) Effect of UVRAG overexpression on HCV replicon cells. HCV replicon cells were transfected with the control vector or flag-UVRAG plasmid for two days. Cells were then lysed for Western-blot analysis. (H) Relative HCV RNA levels as measured by real-time RT-PCR. HCV replicon cells transfected with either the control vector or the flag-UVRAG plasmid for two days were lysed for quantification of HCV RNA by real-time RT-PCR. In (F) and (H), *, *p*<0.05.

To further determine how Rubicon affected HCV replication, we analyzed the HCV subgenomic RNA replicon, which expressed only the HCV nonstructural proteins NS3-NS5B and could induce autophagosomes [3,5]. We first analyzed the protein levels of p62, LC3-II, Rubicon and URVAG in stable HCV subgenomic RNA replicon cells that we had previously established in our laboratory [5]. As shown in Fig. 6A, the HCV replicon cells had increased expression levels of p62, LC3-II, Rubicon and UVRAG. The increases of Rubicon and UVRAG were approximately 1.7-fold and 1.6-fold, respectively. These fold increases were similar to what was observed in HCV-infected cells at 24 hours post-infection, which were approximately 1.6-fold and 1.5-fold for Rubicon and UVRAG, respectively (Fig. 1A). The increase of p62 suggested that that the maturation of autophagosomes in HCV replicon cells was likely inefficient. To test this possibility, we compared the relative levels of p62 in replicon cells and nutrient-starved cells. As shown in Fig. 6B, in contrast to the HCV RNA replicon, nutrient starvation reduced the p62 level in a time-dependent manner and had no apparent effect on Rubicon whereas the p62 level was increased in replicon cells. To further confirm the Western-blot results, we treated cells with Lysotracker-red, which stained for lysosomes. As our Huh7 and replicon cells expressed GFP-LC3, we were able to analyze the relative populations of autophagosomes (i.e., GFP-LC3 puncta that did not colocalize with lysosomes) and autolysosomes (i.e., GFP-LC3 puncta that colocalized with lysosomes). As shown in Fig. 6C, the control Huh7-GFP-LC3 cells displayed few GFP-LC3 puncta, which were induced after nutrient starvation. Approximately 60% of these GFP-LC3 puncta colocalized with lysosomes (Fig. 6D), indicative of autolysosomes. The HCV replicon cells also had a high level of GFP-LC3 puncta, but in contrast, only approximately 30% of these puncta colocalized with lysosomes. These results were consistent with the Western-blot results shown in Fig. 6B and confirmed that the maturation of autophagosomes was inefficient in replicon cells, comparing with cells that were nutrient starved.

To test whether Rubicon also inhibited the maturation of autophagosomes in replicon cells, we suppressed the expression of Rubicon with its siRNA. As shown in Fig. 6E, the Rubicon siRNA reduced the p62 level in naive Huh7 cells. This result indicated that the depletion of Rubicon could facilitate the maturation of autophagosomes during basal autophagy. The suppression of Rubicon expression with its siRNA also reduced the levels of p62 and LC3-II in HCV replicon cells, indicating that the inhibition of Rubicon expression could also facilitate the maturation of autophagosomes and the degradation of p62 in replicon cells. The inhibition of Rubicon expression in replicon cells led to the reduction of NS5A (Fig. 6E) and the HCV RNA level (Fig. 6F). In contrast, the suppression of UVRAG expression with its siRNA did not significantly affect the levels of p62, LC3-II and HCV NS5A in replicon cells (S5B Fig.). This lack of apparently effect of UVRAG might be due to the dominant inhibitory effect of Rubicon on the maturation of autophagosomes in replicon cells and thus the depletion of UVRAG did not further inhibit the maturation of autophagosomes (S5B Fig.). The over-expression of UVRAG in replicon cells, however, significantly reduced p62, LC-II and HCV NS5A protein levels as well as the HCV RNA level (Fig. 6G and 6H). These results indicated that the over-expression of UVRAG could overcome the inhibitory effect of Rubicon on the maturation of autophagosomes in HCV replicon cells and further confirmed a negative role of UVRAG on HCV RNA replication.

Rubicon may enhance HCV replication via enhancing viral protein translation or viral RNA replication. To distinguish between these two possibilities, we transfected Huh7 cells with a control siRNA or the Rubicon siRNA. These cells were then transfected with a DNA plasmid that expressed a bicistronic HCV RNA, which encoded the renilla luciferase at its 5’-end and the firefly luciferase at its 3’-end. In this bicistronic RNA, the translation of renilla luciferase was cap-dependent whereas the translation of the firefly luciferase was mediated by the HCV IRES. As shown in S6 Fig., the suppression of Rubicon expression had no significant effect on the HCV IRES activities. This result indicated that Rubicon enhanced HCV replication not by increasing the HCV IRES activity but rather, by facilitating HCV RNA replication.

### Induction of Rubicon expression by HCV NS4B

**Fig 7:**
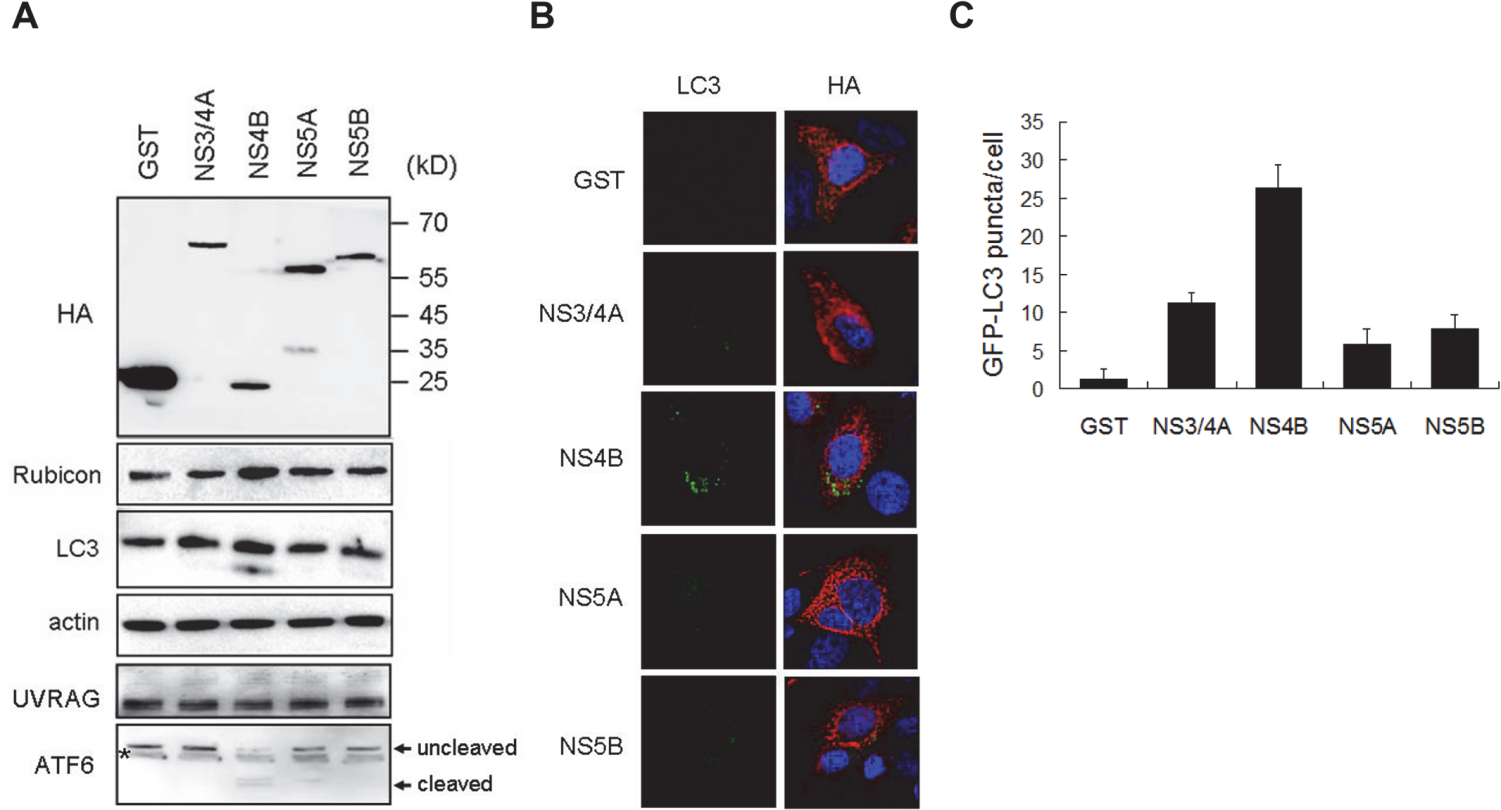
Effects of HCV nonstructural proteins on the induction of Rubicon and autophagosomes. (A) Western-blot analysis of Huh7 cells transfected with the expression plasmids of HA-tagged GST and various HCV nonstructural proteins. Cells were lysed 48 hours after transfection for analysis. The localizations of molecular weight markers are indicated. The asterisk denotes a nonspecific protein band. (B) Analysis of GFP-LC3 puncta in stable Huh7 cells that expressed GFP-LC3. Cells were transfected with various expression plasmids for 48 hours and immunostained with the anti-HA antibody (red color). GFP-LC3 puncta were apparent in cells that expressed HCV NS4B. (C) The average number of GFP-LC3 puncta per cell shown in (B). The results represent the mean of >30 cells.

As the HCV subgenomic RNA replicon, which expressed HCV NS3, NS4A, NS4B, NS5A and NS5B, was sufficient to induce Rubicon, we tested whether any of these HCV gene products could induce Rubicon. We transfected Huh7 cells with the plasmids that expressed GST, NS3/4A, NS4B, NS5A and NS5B, which were all HA-tagged. The GST protein served as the negative control. As shown in Fig. 7A, the expression of GST, NS3/4A, NS5A and NS5B had no significant effect on the expression of Rubicon and the lipidation of LC3. However, the expression of NS4B clearly increased the protein levels of Rubicon and LC3-II. None of the HCV proteins tested had any apparent effect on UVRAG. Interestingly, NS4B could also induce the cleavage of ATF6, an important marker of the unfolded protein response (UPR), suggesting a possible role of UPR in the induction of Rubicon by NS4B. We also transfected these expression plasmids into stable GFP-LC3 Huh7 cells for the analysis of induction of autophagic puncta. Again, as shown in Fig. 7B and 7C, the expression of NS4B had the most prominent effect on the induction of autophagic puncta, although other HCV proteins were also able to induce autophagic puncta, albeit to a lesser degree. These results indicated that the HCV NS4B protein was sufficient to induce the expression of Rubicon to inhibit the maturation of autophagosomes.

## Discussion

**Fig 8:**
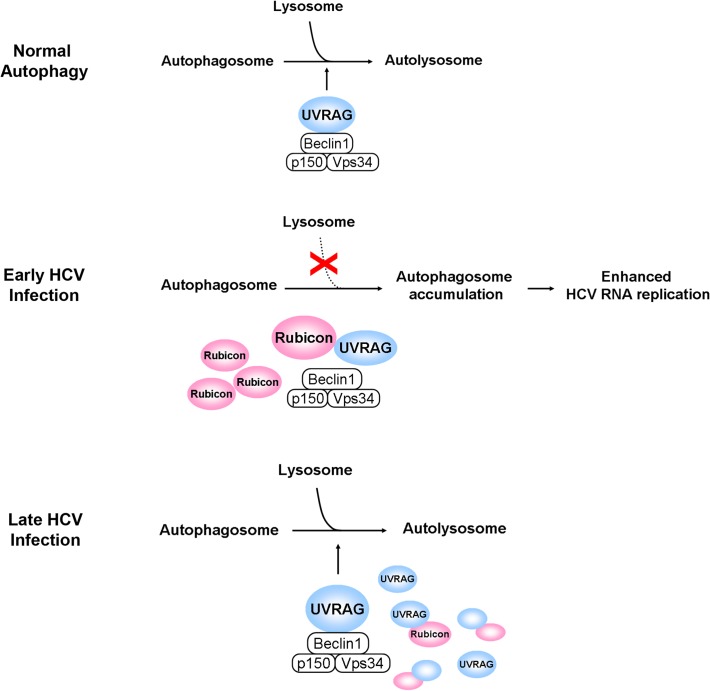
Model for the roles of Rubicon and UVRAG in the maturation of autophagosomes in HCV-infected cells. In the normal autophagic pathway, UVRAG, in complex with Beclin-1, p150 and Vps34, facilitates the fusion between autophagosomes and lysosomes to form autolysosomes. The induction of Rubicon by HCV in the early stage of infection inhibits the UVRAG activity and the fusion between autophagosomes and lysosomes. This leads to the accumulation of autophagosomes, which enhance HCV RNA replication. The induction of UVRAG in the late stage of HCV infection overcomes the inhibitory effect of Rubicon and results in the maturation of autophagosomes. In the model illustrated, the effect of HCV on the initiation of autophagy is not addressed.

It has previously been shown that HCV infection can induce autophagy. In this report, we demonstrated that the induction of the autophagic response by HCV was rapid and could be detected as early as six hours post-infection (Fig. 1). This induction of autophagic response began with the accumulation of autophagosomes, which matured inefficiently at the early time points of HCV infection (Fig. 1C). This inefficient maturation of autophagosomes was due to the induction of Rubicon by HCV, as the suppression of Rubicon expression facilitated the maturation of autophagosomes (Fig. 2), and the over-expression of Rubicon had the opposite effect (Fig. 3). These results indicated that the accumulation of autophagosomes in the early stage of HCV infection was at least partially due to the inhibition of the maturation of autophagosomes (i.e., the reduction of the autophagic “off-rate”). In contrast to the early time points, the maturation of autophagosomes was efficient at 48 hours post-infection (Fig. 1). The increased maturation efficiency of autophagosomes at this time point was apparently due to the induction of UVRAG by HCV, which overcame the suppressive effect of Rubicon, as the over-expression of UVRAG enhanced the maturation of autophagosomes in HCV-infected cells (Fig. 4). The model of how HCV regulates the maturation of autophagosome at different stage of infection is illustrated in Fig. 8. Note that in spite of the increase of the maturation efficiency of autophagosomes at the latter time point, the overall number of autophagic vacuoles remained high at that time point (Fig. 1B and 1C). It is conceivable that this was due to the higher autophagic initiation rate (i.e., the autophagic “on-rate”) caused by the higher expression level of UVRAG, which is known to enhance the initiation of autophagy [18].

Our studies also demonstrated that Rubicon enhanced HCV replication whereas UVRAG inhibited HCV replication (Fig. 5). By using cells that harbored the HCV subgenomic RNA replicon, we demonstrated that the effect of Rubicon on HCV was most likely on HCV RNA replication (Fig. 6 and S6 Fig.). As our previous studies indicated that autophagosomal membranes could serve as the sites for HCV RNA replication, it is likely that Rubicon enhanced HCV RNA replication by increasing the pool of autophagosomes in infected cells. This could also explain why the over-expression of UVRAG, which reduced the pool of autophagosomes by facilitating their maturation, would reduce HCV RNA replication. Our results thus are consistent with the model that HCV induces the expression of Rubicon to enhance the accumulation of autophagosomes, which serve as sites for HCV RNA replication in the early stage of HCV replication. However, due to the extensive reorganization of membrane structures in the later stage of infection, autophagosomes may be replaced by other membrane structures such as the smaller double membrane vesicles (DMVs) [29,30], which may also be derivatives of autophagosomes due to their structural similarities, and become dispensable for HCV RNA replication. This model will also explain why previous studies indicated that autophagy is only important for HCV replication in the early stage, but not in the later stage, of infection [4].

We had also studied the mechanism of Rubicon induction by HCV. Our results indicated that HCV NS4B was sufficient to induce its expression and the accumulation of autophagosomes (Fig. 7). HCV NS4B has previously been shown to interact with Rab5 and Vps34 to regulate autophagy [8,31]. It is unclear whether these activities of NS4B contributed to its effect on Rubicon. Previous studies indicated that HCV induced the autophagic response via the UPR [5,7,11]. It is interesting that NS4B by itself was sufficient to induce the cleavage of ATF6, an important event of the UPR (Fig. 7A). This result is consistent with a previous report, which indicated that HCV NS4B by itself could induce the UPR [32]. It is conceivable that NS4B might induce Rubicon via the UPR, which could activate a number of transcription factors including ATF6. Some of these transcription factors might be involved in the up-regulation of the Rubicon gene, as our results indicated that the induction of Rubicon by HCV was likely a transcriptional event (S3 Fig.).

Many viruses can perturb the autophagic pathway to enhance their replications and, similar to HCV, some viruses can inhibit the fusion between autophagosomes and lysosomes [33–38]. For example, poliovirus and coxsakievirus B3 induce autophagosomes to support its replication [39,40]. Rotavirus also blocks the maturation of autophagosomes and use the autophagic vacuoles for the transport of viral proteins from ER to viroplasms where the viral genome replication and particle assembly take place [36]. Dengue virus-2 (DENV-2), however, uses amphisomes, an intermediate autophagic vacuole prior to the formation of autolysosomes, for its protein translation and RNA replication [41]. In contrast to DENV-2, DENV-3, a different DENV serotype, uses both amphisomes and autolysosomes for its replication [42]. Kaposi's sarcoma-associated herpesvirus (KSHV) can also inhibit the maturation of autophagosomes to enhance its replication [43]. Interestingly, KSHV inhibits the maturation of autophagosomes by using its K7 protein to bind to Rubicon. This previous observation on KSHV K7 indicates that the effect of HCV on Rubicon and the regulation of autophagosomal maturation is not unique. It is likely that this may be a mechanism frequently exploited by viruses to control the autophagic pathway in their host cells for their replication.

## Materials and Methods

### Cell lines, the HCV virus stock and DNA plasmids

Huh7 and its derivative Huh7.5 (gift of Dr. Charles Rice, Rockefeller University) are human hepatoma cell lines [5]. They were maintained at 37°C in Dulbecco's modified Eagle medium (DMEM) supplemented with 10% fetal bovine serum (FBS) and nonessential amino acids. Huh7N1b replicon cells harboring an HCV subgenomic RNA replicon were maintained in the same medium containing 0.8 mg/ml G418 (Sigma-Aldrich). Depending on the experiments, cells might be nutrient-starved in Hank’s balanced salt solution (HBSS) for 2 or 6 hours before being harvested. Huh7.5 cells that stably expressed mRFP-GFP-LC3 were established by transfecting the cells with the mRFP-GFP-LC3 expression plasmid provided by Dr. David Ann. Twenty-four hours after transfection, cells were selected with DMEM containing G418 (800 μg/mL). Stable cell colonies were pooled and maintained in DMEM supplemented with 10% FBS and G418 (500 μg/mL). All of our infection studies were conducted using a variant of the HCV JFH1 isolate. This variant replicated more efficiently in Huh7.5 cells than the original JFH1 isolate [27]. The bicistronic reporter plasmid pRL-HL, which produced the bicistronic mRNA that expressed the renilla luciferase via a cap-dependent translation mechanism and the firefly luciferase using HCV IRES, had been described before [26]. The expression plasmids for HA-tagged GST, NS3/4A, NS4B, NS5A and NS5B had also been described before [44].

### DNA transfection

The plasmid DNA was mixed with the BioT transfection reagent (Bioland) in serum-free DMEM to a final concentration of 2μg/mL per the manufacturer’s protocol. This transfection mixtures was incubated at room temperature for 20 min prior to inoculation into cells. Two days after transfection, cells were harvested for further studies.

### Antibodies

The primary antibodies used in this study included the rabbit anti-Rubicon antibody (Abcam), rabbit anti-UVRAG antibody (Sigma-Aldrich), rabbit anti-p62 antibody (Cell Signaling), mouse anti-HCV NS5A monoclonal antibody (Millipore), rabbit anti-LC3 antibody (MBL), and rabbit anti-core antibody [45]. Proteins were extracted from cell lysates for Western blotting using the M-PER mammalian protein extraction reagent (Thermo Scientific) following the manufacturer’s protocol.

### siRNA knockdown of Rubicon and UVRAG

For the siRNA knockdown experiment, siRNAs (100 μM) against Rubicon (SASI_Hs02_00346051) and UVRAG (SASI_Hs01_00113688) (Sigma-Aldrich) were transfected into cells using Lipofectamine RNAiMAX (Invitrogen) in Opti-MEM (Invitrogen). Briefly, 4 × 10^4^ cells seeded in a 35-mm dish were transfected with 2 μl of siRNAs (100 μM each) for 6 h and then the transfection mixture was replaced by fresh DMEM. Replicon cells were harvested 48 hours post-transfection for protein and RNA analyses, and Huh7.5 cells were infected with HCV using a multiplicity of infection (m.o.i.) of 1. Infected cells were then harvested at various time points for further analysis.

### Focus-formation assay for HCV titration

Huh7.5 cells were seeded onto the 8-well chamber slide (2x10^4^ cells/well) and inoculated with serially diluted HCV the next day. Forty-eight hours after infection, cells were washed with phosphate-buffered saline (PBS) and fixed with 3.7% paraformaldehyde for 15 minutes. Cells were then stained with the rabbit anti-core primary antibody for 2 hours and then with the Alexa-488-conjugated goat anti-rabbit secondary antibody for 2 more hours. After washing, cells on the slide were mounted with VectorShield with DAPI. The HCV core-positive cells were counted under the microscope for titration

### Western-blot analysis

Cells were washed with PBS and lysed with M-PER Mammalian Protein Extraction Reagent (Thermo). After centrifugation to remove cell debris, cell lysates were subjected for SDS-PAGE electrophoresis. After the semi-wet transfer, the membrane was blocked with 5% skim milk for 1 hour and incubated with the primary antibody overnight. After three washes with PBS containing 1% Tween 20 (PBST), the membrane was incubated with the HRP-conjugated secondary antibody for 1 hour. After further washes with PBST, chemiluminescent substrates (Pierce) were applied on the membrane, and the image was captured using the LAS-4000 imaging system (FujiFilm).

### Quantitative and semi-quantitative RT-PCR

Total RNA was isolated from Huh7.5 cells using TRIZOL (Invitrogen) following the manufacturer’s protocol. RNA thus isolated was reverse transcribed with SuperScript II Reverse Transcriptase (Invitrogen) and oligo(d)T primers in the presence of RNasin (Promega). Gene-specific primers were used to amplify cDNA. qPCR was performed using the Taqman PCR core reagent system (Roche) and analyzed by the Fast Real-Time PCR system (ABI). Semi-quantitative PCR was performed using GoTaq Green Master Mix (Promega) and the products were analyzed by DNA gel electrophoresis.

### Immunofluorescence staining and microscopy

For Lysotracker staining, cells were incubated in growth media containing 50 nM LysoTracker Red DND-99 (Invitrogen, Carlsbad, CA) at 37°C for 1.5 hours. After the incubation, cells were rinsed with phosphate-buffered saline (PBS) and then fixed with 3.7% formaldehyde. Cells were permeabilized with PBS containing 0.1% saponin, 1% bovine serum albumin (BSA) and 0.05% sodium azide for 5 minutes, and incubated with antibodies for immunofluorescence microscopy. Cover-slips were mounted in VectorShield (Vector) containing DAPI, which stained the DNA. Images were acquired with the Keyence All-in-one fluorescence microscope. The colocalization coefficient, which measures the fraction of green fluorescent protein (GFP) pixels that are also positive for LysoTracker-red, was performed on randomly selected cells (>50) using the Image J imaging software.

## Supporting Information

S1 Fig Infection analysis of Huh7.5 cells by HCV. (A) Huh7.5 cells were infected by the HCV JFH-1 variant (m.o.i. = 1) and stained for the HCV core protein (green color) at 48 hours post-infection. Mock-infected cells were used as the control. Nuclei were stained with DAPI (blue color). (B) Stable Huh7.5 cells that expressed the mRFP-GFP-LC3 were infected with HCV (m.o.i. = 1). The HCV core protein was stained with the anti-core antibody (blue color). The inset (boxed) was enlarged and shown to the right.
(TIF)

S2 Fig Infection analysis of Huh7 cells by HCV. Huh7 cells were infected by HCV (m.o.i. = 1) and lysed at different time points after infection for Western-blot analysis. Numbers under Rubicon and UVRAG panels indicated the expression levels of these proteins relative to the mock-infected control (i.e., 0 hours p.i.)
(TIF)

S3 Fig Induction of Rubicon and UVRAG RNAs by HCV. Huh7.5 cells infected by HCV were lysed at different time points for the isolation of total cellular RNA. The levels of Rubicon and UVRAG RNAs were then analyzed by the semi-quantitative RT-PCR. The actin RNA was also analyzed to serve as an internal control. Numbers under the Rubicon and UVRAG panels indicated the fold increase of the RNA level relative to the 0 hour.
(TIF)

S4 Fig Immunofluorescence analysis of Flag-tagged Rubicon and UVRAG. Huh7.5 cells were transfected with the control vector or the expression plasmid of Flag-tagged Rubicon or Flag-tagged UVRAG. The transfection efficiency was then analyzed by immunofluorescence staining using the anti-Flag antibody.
(TIF)

S5 Fig Suppression of UVRAG expression in HCV-infected Huh7.5 cells and HCV replicon cells. (A) Huh7.5 cells were transfected with either the control siRNA (NC) or the UVRAG siRNA (UV) for 48 hours followed by HCV infection for 24 hrs. Cells were then lysed for Western-blot analysis. (B) HCV subgenomic replicon cells were transfected with either the control siRNA or the UVRAG siRNA for 48 hours. Cell lysates were then subjected to Western-blot analysis.
(TIF)

S6 Fig Effect of Rubicon on HCV IRES activity. Huh7 cells were transfected with either the control siRNA (siNC) or the Rubicon siRNA (siRb) for two days followed by the transfection of the reporter plasmid pHL-RL. pHL-RL expressed a bicistronic RNA (see illustration on the top of the Fig), which encoded the renilla luciferase at the 5’-end and the firefly luciferase at the 3’-end. The translation of the renilla luciferase was cap-dependent whereas that of the firefly luciferase was under the control of the HCV IRES. The relative HCV IRES activity was determined by dividing the firefly luciferase activity of siRb-transfected cells with that of siNC-transfected cells after the normalization of the firefly luciferase activity against the renilla luciferase activity. n.s., statistically not significant.
(TIF)
