# Supplementary figures and images for: HCV Induces the Expression of Rubicon and UVRAG to Temporally Regulate the Maturation of Autophagosomes and Viral Replication
Source: PLoS Pathog. 2015 Mar 25;11(3):e1004764. doi: 10.1371/journal.ppat.1004764 (PMC4373777; doi:10.1371/journal.ppat.1004764)

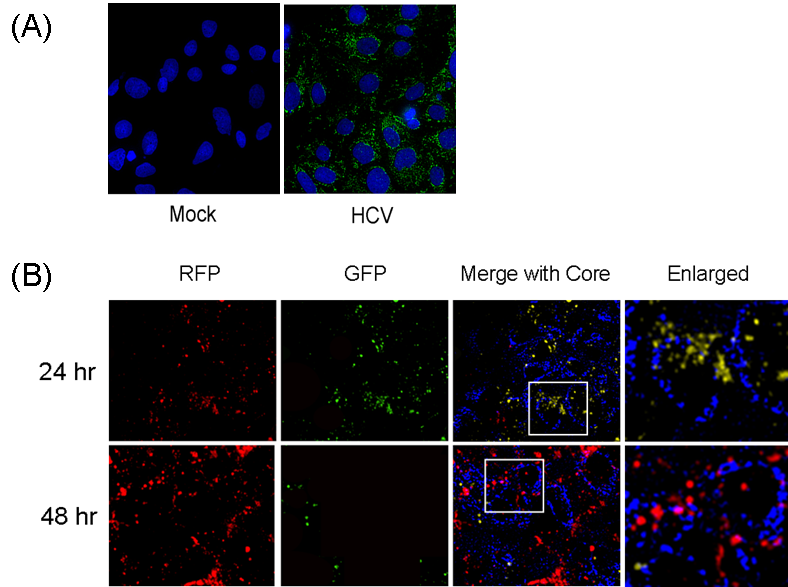

Supplement: S1 Fig — (A) Huh7.5 cells were infected by the HCV JFH-1 variant (m.o.i. = 1) and stained for the HCV core protein (green color) at 48 hours post-infection. Mock-infected cells were used as the control. Nuclei were stained with DAPI (blue color). (B) Stable Huh7.5 cells that expressed the mRFP-GFP-LC3 were infected with HCV (m.o.i. = 1). The HCV core protein was stained with the anti-core antibody (blue color). The inset (boxed) was enlarged and shown to the right. (TIF) [file ppat.1004764.s001.tif]

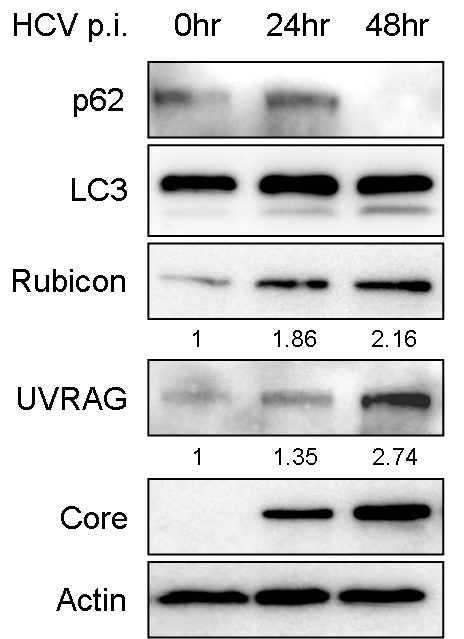

Supplement: S2 Fig — Huh7 cells were infected by HCV (m.o.i. = 1) and lysed at different time points after infection for Western-blot analysis. Numbers under Rubicon and UVRAG panels indicated the expression levels of these proteins relative to the mock-infected control (i.e., 0 hours p.i.) (TIF) [file ppat.1004764.s002.tif]

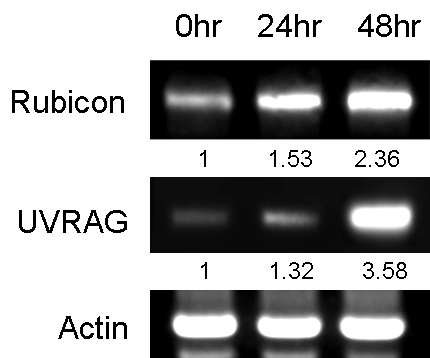

Supplement: S3 Fig — Huh7.5 cells infected by HCV were lysed at different time points for the isolation of total cellular RNA. The levels of Rubicon and UVRAG RNAs were then analyzed by the semi-quantitative RT-PCR. The actin RNA was also analyzed to serve as an internal control. Numbers under the Rubicon and UVRAG panels indicated the fold increase of the RNA level relative to the 0 hour. (TIF) [file ppat.1004764.s003.tif]

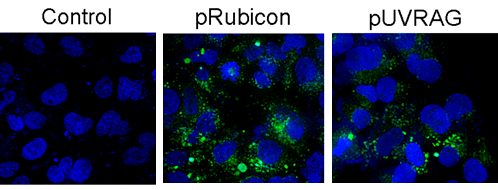

Supplement: S4 Fig — Huh7.5 cells were transfected with the control vector or the expression plasmid of Flag-tagged Rubicon or Flag-tagged UVRAG. The transfection efficiency was then analyzed by immunofluorescence staining using the anti-Flag antibody. (TIF) [file ppat.1004764.s004.tif]

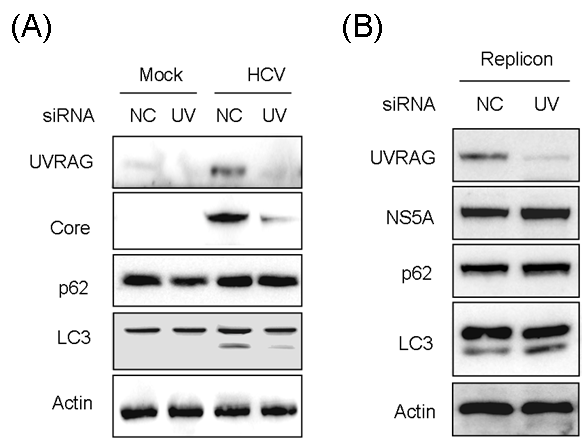

Supplement: S5 Fig — (A) Huh7.5 cells were transfected with either the control siRNA (NC) or the UVRAG siRNA (UV) for 48 hours followed by HCV infection for 24 hrs. Cells were then lysed for Western-blot analysis. (B) HCV subgenomic replicon cells were transfected with either the control siRNA or the UVRAG siRNA for 48 hours. Cell lysates were then subjected to Western-blot analysis. (TIF) [file ppat.1004764.s005.tif]

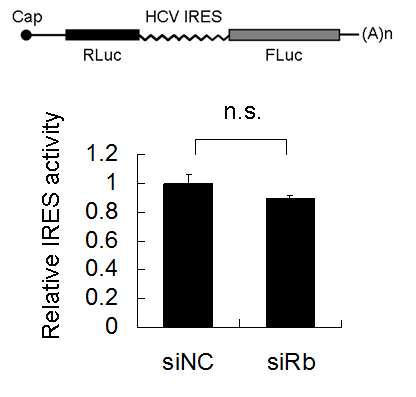

Supplement: S6 Fig — Huh7 cells were transfected with either the control siRNA (siNC) or the Rubicon siRNA (siRb) for two days followed by the transfection of the reporter plasmid pHL-RL. pHL-RL expressed a bicistronic RNA (see illustration on the top of the Fig), which encoded the renilla luciferase at the 5’-end and the firefly luciferase at the 3’-end. The translation of the renilla luciferase was cap-dependent whereas that of the firefly luciferase was under the control of the HCV IRES. The relative HCV IRES activity was determined by dividing the firefly luciferase activity of siRb-transfected cells with that of siNC-transfected cells after the normalization of the firefly luciferase activity against the renilla luciferase activity. n.s., statistically not significant. (TIF) [file ppat.1004764.s006.tif]
